# Temozolomide (TMZ) in the Treatment of Glioblastoma Multiforme—A Literature Review and Clinical Outcomes

**DOI:** 10.3390/curroncol31070296

**Published:** 2024-07-12

**Authors:** Marcin Jezierzański, Natalia Nafalska, Małgorzata Stopyra, Tomasz Furgoł, Michał Miciak, Jacek Kabut, Iwona Gisterek-Grocholska

**Affiliations:** 1 Faculty of Medicine, Silesian Medical University, 41-800 Zabrze, Poland; s81185@365.sum.edu.pl (N.N.); s81569@365.sum.edu.pl (M.S.); s80882@365.sum.edu.pl (T.F.); 2 Faculty of Medicine, Wroclaw Medical University, 50-367 Wroclaw, Poland; michal.miciak@student.umw.edu.pl; 3 Department of Oncology and Radiotherapy, Silesian Medical University, Ceglana 35, 40-514 Katowice, Poland; jkabut@sum.edu.pl (J.K.); igisterek@sum.edu.pl (I.G.-G.)

**Keywords:** temozolomide: glioblastoma multiforme, anticancer therapy, effectiveness, overall survival

## Abstract

Glioblastoma multiforme (GBM) is one of the most aggressive primary tumors of the central nervous system. It is associated with a very poor prognosis, with up to half of patients failing to survive the first year after diagnosis. It develops from glial tissue and belongs to the adult-type diffuse glioma group according to the WHO classification of 2021. Therapy for patients with GBM is currently based on surgical resection, radiation therapy, and chemotherapy, but despite many efforts, there has been minimal progress in tumor management. The most important chemotherapeutic agent in the treatment of this tumor is temozolomide (TMZ), a dacarbazine derivative that presents alkylating activity. It is usually administered to patients concurrently with radiation therapy after surgical resection of the tumor, which is defined as the Stupp protocol. Temozolomide demonstrates relatively good efficacy in therapy, but it could also present with several side effects. The resistance of GBM to the drug is currently the subject of work by specialists in the field of oncology, and its use in various regimens and patient groups may bring therapeutic benefits in the future. The aim of this review paper is to summarize the relevance of TMZ in the treatment of GBM based on recent reports.

## 1. Introduction

Glioblastoma multiforme (GBM) is the most common primary malignant brain tumor in adults [1,2]. It represents approximately 57% of all gliomas and 48% of all primary central nervous system (CNS) malignancies [3]. It is considered the most aggressive form of primary intracranial tumor and is associated with a poor prognosis [4]. Very rarely, it can develop as a result of transformation from other CNS tumor types, for example, anaplastic pleomorphic xanthoastrocytoma [5]. The incidence of GBM varies depending on the report, from 3.19 cases per 100,000 person-years to 4.17 per 100,000 person-years [6]. The median age at diagnosis is 65, and the incidence rate is the highest in the 75- to 84-year-old age group. GBM is 1.58 times more common in males. Patients with GBM have a very poor prognosis with only 15 months of median overall survival, and survival of more than three years (3–5% of patients) is already defined as long-term [4,6]. Over the past 30 years, there has only been an insignificant improvement in survival rates for patients with GBM [1,7]. The histopathological features of GBM include a diffuse neoplastic infiltration of neural tissue with a necrotic core and astroglial-like cells (“angular” nucleus, euchromatin), and vascular proliferation or pseudopalisading necrosis with mitoses [1]. CNS tumors are classified according to the WHO classification. This classification has undergone numerous changes over the past years. At present, the 2021 classification is the most current version. GBM also includes IDH-wt and IDH-mutant GBM subtypes. Before 2021, this division did not exist. Only G3 and G4 grading was considered, where G4 was regarded as GBM. The place of gliomas in the 2021 WHO classification of CNS tumors is presented in Table 1. The structure of the blood-brain barrier (BBB), the inhibitory tumor microenvironment, and the heterogeneity of the tumor provide glioma cells with an advantage, leading to the reduced efficacy of chemotherapeutics, targeted therapies, and immunotherapy [1,8]. The current standard of care for patients with GBM includes surgical resection, radiation therapy, and chemotherapy. The first-line drug for the treatment of GBM is the chemotherapeutic agent temozolomide [9,10]. The purpose of this paper is to describe the place of this anticancer drug and its efficacy in GBM therapy based on recent clinical trials.

## 2. Materials and Methods

Online databases were searched: PubMed, PubMed Central, and GoogleScholar. We used keywords related to “temozolomide”, “glioblastoma multiforme”, “GBM antineoplastic therapy”, and “temozolomide efficacy” and retrieved 35 articles from the last eight years for the final analysis. It is noteworthy that the WHO classification of CNS tumors was modified during the analyzed period. We decided not to exclude articles published before 2021, where both tumor subtypes (IDH-wt and IDH-mutant) were included under the phrase “GBM”, which are differentiated in the current classification. In our study, the phrase “GBM” refers to both the former and 2021 classifications due to the similar therapeutic approach with TMZ. The use of such search criteria was also intended to provide a more comprehensive review. Articles were selected after analyzing their abstracts, and those that matched and described the topic in a proper way were used. These included original articles, reviews, and meta-analyses on the latest scientific reports in this field of neuro-oncology. The collected information has been divided into proposed sections, which present the results of scientific studies on groups of patients appropriately selected by the authors and comprehensively reviewed.

## 3. Results

Temozolomide (TMZ) is a derivative of dacarbazine, which is an anticancer drug due to its alkylating properties. It is absorbed into the blood very effectively after oral administration and has the ability to cross the blood-brain barrier (BBB). The drug was approved for the treatment of GBM in 2005 and is also used to treat other solid tumors, including advanced neuroendocrine tumors [12,13,14].

### 3.1. TMZ Side Effects

TMZ is well tolerated by patients, and the side effects, in general, remain moderate. Only 15% of patients discontinue treatment due to the intolerable side effects of TMZ. The most common side effects experienced by patients are fatigue, nausea, vomiting, and myelosuppression. The most severe adverse effects during TMZ therapy are hematologic complications, which include neutropenia, thrombocytopenia, lymphopenia, and leukopenia. A 2015 meta-analysis analyzed the incidence of grade III–IV hematologic complications (Grade I are mild, Grade II are moderate, Grade III are severe, and Grade IV are life-threatening adverse events) among patients undergoing four different TMZ therapeutic regimens for high-grade GBM. The analysis showed a significant difference in the incidence of grade III–IV lymphopenia (Grade III: 200–499/μL, Grade IV: <200/μL in absolute lymphocyte count), which was 76.5% for the standard regimen and significantly higher than for the other three therapeutic regimens. The estimated incidence of neutropenia, thrombocytopenia, and leukopenia oscillated between 5.7% and 9.7% depending on the regimen [15]. Comprehensive hematologic evaluation is used during therapy, which is performed around day 22 of the 28-day cycle. TMZ can cause liver damage, even though this is a rare complication of treatment, but liver function monitoring is also highly recommended [16,17,18,19,20].

### 3.2. TMZ Resistance

Although TMZ remains the primary chemotherapeutic agent for GBM, half of the patients develop resistance to treatment [21]. The most well-known mechanism is the effect of O6-methylguanine DNA methyltransferase (MGMT) activity. This is one of the enzymes responsible for DNA repair and the occurrence of unique resistant glioma stem cell populations. In recent years, a number of other potential mechanisms affecting the resistance of GBM to TMZ have been recognized. These include other DNA repair mechanisms like the mismatch repair pathway (MMR) or base excision repair (BER), abnormal signaling pathways, autophagy, epigenetic modifications, microRNAs, and extracellular vesicle production [22]. Some genes potentially responsible for resistance among GBM cells have also been identified. One of these is the KDMA5 gene, which encodes histone demethylase. Banelli B. et al. noted a clear correlation between the expression of this gene and the occurrence of TMZ resistance in GBM cells and called it a “key” gene in the development of this phenomenon [23]. Another gene described is HDAC6, which encodes histone deacetylase. This enzyme regulates many biological processes, including carcinogenesis, through its deacetylase properties and its ability to bind ubiquitin. Wang Z. et al. observed that there is an increased expression of HDAC6 in glioma cells. HDAC6 promotes glioma cell proliferation and confers TMZ resistance to glioma cells, mainly by stabilizing and activating EGFR. In contrast, Kim G.W. et al. showed that HDAC6 inhibition correlates with increased levels of MSH2 and MSH6—key DNA repair proteins—and decreased expression of MGMT [24,25]. In TMZ-resistant GBM cell populations, morphological changes can also be observed in addition to biochemical changes. Tiek D.M. et al. conducted a study in which the in vitro phenotype of two TMZ-resistant GBM cell lines was compared with that of chemotherapeutic-sensitive lines from the control group. The authors showed that the development of resistance was accompanied by changes such as increased proliferation and migration, an increase in the frequency of chromosomal aberrations, and the secretion of cytosolic lipids [26]. Acquired resistance to TMZ seems to be the main reason for the short median survival of GBM patients, thus understanding the mechanisms responsible for it is a key issue for the effective treatment of GBM. Potential strategies to overcome GBM resistance include combination therapies with TMZ-enhancing agents, targeted therapies that act on both the main mass of the tumor and tumor stem cells, which can play a key role in tumor recurrence, anti-vascular therapies, or immunotherapy [27].

### 3.3. The Role of TMZ in GBM Therapy and Therapeutic Regimens

**Figure 1:**
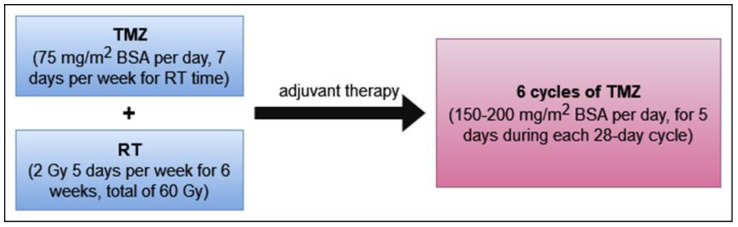
Stupp standard protocol for the treatment of GBM after tumor surgical resection.

Currently, the most commonly used regimen in the treatment of GBM is the Stupp protocol that involves the combination of radiotherapy and TMZ chemotherapy after surgical resection of the tumor. The details are summarized in Figure 1. Stupp’s study in a group of GBM patients showed that the use of TMZ in combination with radiotherapy prolongs survival by 2.5 months compared to radiotherapy alone. The median overall survival (OS) time increases from 12.1 months to 14.6 months. The 2-year OS rate also increases from 10.4% to 26.5% [28].

After standard combined chemoradiotherapy and adjuvant chemotherapy, most patients relapse within 6 months. There is currently no recognized systemic treatment regimen for second-line therapy. However, alkylating chemotherapy is commonly used. Lomustine, carmustine, and re-treatment with TMZ are potential options, although the benefits are insignificant and probably only applicable to the group of patients with MGMT promoter methylation [29]. TMZ remains the main drug in monotherapy. Nevertheless, there exist studies comparing the efficacy of its application together with the aforementioned carmustine. Several authors report achieving a minor increase in OS with a local implantation of carmustine in the resection cavity; however, it would be beyond the scope of this review [30]. In a meta-analysis of 33 studies involving 1760 patients with recurrent tumor, the standard regimen and three common dose-escalated TMZ regimens for the treatment were included: 150–200 mg/m^2^ for 1–5 days every 28 days (standard regimen); 100–150 mg/m^2^ for days 1–7 and 15–21 in 28-day cycles; continuous daily dosing at 40–50 mg/m^2^; and 75–100 mg/m^2^ for days 1–21 in each 28-day cycle. The 7-day treatment/7-day break regimen was shown to be significantly superior in terms of progression-free survival (PFS) at 6 months (34.8%) and 12 months (15.5%) compared to the standard 5-day regimen (23.1% and 7.5%, respectively). Furthermore, the regimen of 21 days of TMZ administration and 7 days off had a significantly longer OS rate at 6 months (73.6%; 95% CI 63.4–81.8%) and 12 months (40.6%; 95% CI 32.6–48.6%) than the standard regimen. The lymphopenia toxicity rate for the standard regimen was 76.5% (95% CI 45.5–92.7%), the highest of all four regimens studied. The researchers suggest that alternative TMZ dosing regimens in recurrent GBM have benefits for both survival and tumor response. For these reasons, treatment of recurrent tumor should be personalized and actively monitored, especially in patients with concomitant hematologic diseases [15].

### 3.4. TMZ versus Radiation Therapy

The phase III CATNON trial included adult patients with newly diagnosed anaplastic gliomas without 1p/19q deletion, with molecular features of GBM (they were redesigned as IDH-wt GBM in 2021 according to the WHO classification), who were randomly assigned (1:1:1:1) to radiotherapy alone (RT), radiotherapy in combination with TMZ (RT/TMZ), radiotherapy with TMZ add-on, and both in combination and add-on [31]. Among the 751 patients included in the study, a total of 159 met the WHO 2021 molecular criteria for wild-type IDH (IDH-wt) glioma, and there was no additive effect of TMZ on either OS or PFS. MGMT promoter methylation was a prognostic factor for OS but not for TMZ treatment outcome for either OS or PFS. The study’s authors emphasize that these findings call for a new prospective clinical trial to evaluate the efficacy of TMZ treatment in this patient population [32]. In 2015, Joo J.D. et al. published an evaluation of the efficacy and safety of combined chemoradiotherapy and adjunctive treatment with TMZ in newly diagnosed GBM as a standard treatment protocol in a retrospective study among 71 patients. The response rate to therapy was 41% and the tumor control rate was 80%. In the 67 patients who completed chemoradiotherapy with TMZ, the median OS was 19 months, and the 1- and 2-year OS rates were 78.3% and 41.7%, respectively. Median PFS was 9 months, and 1- and 2-year PFS rates were 33.8% and 14.3%, respectively. Simultaneous chemoradiotherapy with TMZ resulted in grade 3 or 4 hematologic toxicities in 2.8% of patients [33]. In 2020, a meta-analysis examining the effect of RT with adjuvant TMZ on the efficacy of GBM treatment was published, proving that the use of RT/TMZ compared to RT alone in the treatment of GBM was associated with a significant improvement in OS (HR = 0.63). It was further shown that patients with MGMT-methylated tumors benefited the most from RT/TMZ, with a median OS that almost doubled in this group (13.5 months vs. 7.7 months). In both subgroups of patients with the IDH mutation (IDHmt) and IDH-wt, PFS was comparable in those treated with RT and TMZ, and patients with IDHmt tumors displayed a better prognosis than IDH-wt [34].

### 3.5. TMZ in Patients over 65 Years of Age

Perry J.R. et al. performed a study showing that in elderly patients with GBM, the addition of TMZ to hypofractionated RT (40 Gy in 15 fractions) resulted in longer survival compared to RT alone. A total of 562 patients were randomized, with a median age of 73 years. The median OS was longer in the combination treatment group compared to RT alone (9.3 months vs. 7.6 months), and median PFS (5.3 months vs. 3.9 months). The 1- and 2-year survival rates in the group with addition of chemotherapy were 37.8% and 10.4%, and in the RT-alone receiving group: 22.2% and 2.8%, respectively. Among 165 patients with tumors with MGMT promoter methylation, the median OS was 7.7 months in the group receiving stand-alone RT and 13.5 months in the group receiving combination therapy, and among the 189 patients with unmethylated MGMT, OS was 7.9 months and 10 months, respectively. The study showed that the use of TMZ results in a statistically significant improvement in OS and PFS, and older patients tolerate the combination therapy reasonably well [35]. A retrospective cohort analysis of 1652 patients aged ≥65 years with GBM who received ≥10 fractions of RT was published in 2018. In the analysis of patients treated between 2005 and 2009, groups undergoing RT or TMZ/RT alone were selected, while for patients treated from 1995 to 1999, those treated with RT alone were analyzed because chemotherapy was not used in those years. It was shown that among patients treated between 2005 and 2009, the addition of TMZ did not significantly improve survival [36].

### 3.6. TMZ in Prolonged Therapy

In 2017, Xu W. et al. published a meta-analysis on the efficacy and safety of prolonged TMZ therapy (>6 cycles) in patients with highly differentiated GBM. Six studies with 396 patients were included in the OS analysis. The median OS in the Stupp protocol group was 22.93 months, compared to 27.65 months in the prolonged TMZ therapy group. Five studies with a total of 84 adverse effects were included in the safety evaluation. There was no evidence of increased toxicity resulting from prolonged TMZ therapy [37]. A meta-analysis by Alimohammadi E. et al., including seven studies with a total of 1018 patients, showed that the overall survival was higher in the case of the treatment group (>6 cycles TMZ) compared to the control group (6 cycles TMZ) (Z = 2.375, P = 0.018). The lower and upper limits were between 1.002–10.467 months [38]. The GEINO 14-01 trial evaluated 79 patients in the control group and 80 patients in the experimental group who received prolonged treatment up to 12 cycles and showed that increasing the number of cycles did not improve the primary endpoint of 6-month PFS but did increase toxicity. OS reached 20.4 months, including 23.3 months for the control group and 18.2 months for the study group [39]. Gupta T. et al., in a 2022 meta-analysis, compared the effects of prolonged TMZ therapy (>6 cycles) after RT with concurrent TMZ therapy. In five prospective randomized clinical trials, with a total of 358 patients, the authors reported no statistically significant reduction in the risk of progression or death in the study group. The risk of grade ≥3 hematologic toxicity was slightly higher in the study group, while the difference was not statistically significant [40].

## 4. Summary

Temozolomide, an oral alkylating agent, has emerged as a cornerstone in the treatment of various brain tumors, notably GBM. This review provides a comprehensive analysis of the current understanding of TMZ’s role in brain tumor management, encompassing its mechanism of action, clinical efficacy, and resistance mechanisms. TMZ exerts its cytotoxic effects by inducing DNA methylation and alkylation, leading to DNA damage and subsequent apoptosis. The DNA repair enzyme O6-methylguanine-DNA methyltransferase (MGMT) plays a crucial role in counteracting TMZ-induced DNA damage by removing alkyl adducts. Methylation of the MGMT promoter, resulting in its silencing, is associated with an improved response to TMZ therapy and a favorable prognosis in GBM patients. Brain tumors represent a heterogeneous group of neoplasms associated with significant morbidity and mortality. Among these, GBM stands out as the most aggressive and lethal primary brain malignancy. Despite advancements in therapeutic modalities, the prognosis for GBM remains dismal, with a median survival of approximately 12–15 months. TMZ, an oral alkylating agent, has revolutionized the landscape of GBM treatment since its approval in 2005. Its integration into the standard of care, comprising concurrent chemoradiotherapy followed by adjuvant maintenance therapy, has resulted in modest improvements in patient outcomes. TMZ, while effective in treating brain tumors, can cause various side effects. One of the most commonly encountered toxicities is hematological toxicity, manifesting as neutropenia, thrombocytopenia, and anemia. These hematological adverse events can predispose patients to an increased risk of infections and bleeding complications. Patients receiving TMZ-based chemotherapy, particularly those with prolonged lymphopenia, may be at increased risk of *Pneumocystis jiroveci* pneumonia, a potentially severe opportunistic infection. In such cases, prophylactic antibiotics, such as trimethoprim-sulfamethoxazole, may be prescribed to prevent this infection. Gastrointestinal adverse effects, including nausea, vomiting, and anorexia, are frequently reported during TMZ therapy. These symptoms can significantly impact patients’ nutritional status, hydration, and overall well-being, leading to treatment interruptions or dose modifications. Despite its initial efficacy, resistance to TMZ inevitably develops in a subset of GBM patients, leading to treatment failure. Mechanisms of TMZ resistance are multifactorial and encompass both tumor-intrinsic factors, such as MGMT expression and DNA repair capacity, and tumor microenvironment-mediated processes, including hypoxia-induced signaling pathways and immune evasion mechanisms. The development of novel therapeutic strategies to circumvent TMZ resistance represents a pressing need in neuro-oncology. Promising avenues include the targeting of alternative DNA repair pathways, modulation of the tumor microenvironment, and the rational design of combination therapies to enhance TMZ efficacy while minimizing resistance. Furthermore, ongoing translational research efforts aimed at identifying biomarkers predictive of TMZ response hold great potential for personalized treatment approaches in GBM.

## 5. Conclusions

1. GBM is a highly malignant primary tumor of the CNS with some of the lowest long-term survival rates.
2. TMZ has been the basis of GBM chemotherapy for almost 20 years and is part of the standard treatment regimen along with neurosurgery and radiation therapy (Stupp protocol).
3. TMZ is a relatively safe drug.
4. The resistance of GBM cells to TMZ is one of the most important issues in terms of treatment efficacy, determining treatment success.
5. There are many TMZ treatment regimens with efficacy varying on an individual basis; therefore, treatment should be personalized as much as possible.
6. Adjuvant therapy of TMZ after RT is a safe therapeutic option, and its efficacy seems to be higher than that of RT alone, although it largely depends on individual factors such as MGMT promoter status and the histological type of the tumor.
7. Adjuvant TMZ therapy after RT is both safe for patients >65 years of age and improves survival time.
8. Prolonged TMZ therapy (>6 cycles) may result in improved survival of patients with GBM without increasing the frequency of hematologic side effects; however, data on this subject are inconclusive.

## Figures and Tables

**Table 1:** The placement of GBM in the WHO Classification of Tumors of the Central Nervous System, fifth edition, 2021 [11].

2021 WHO Classification. Group 1. Gliomas, Glioneuronal Tumors, and Neuronal Tumors						
Subgroup	Adult-type diffuse gliomas	Pediatric-type diffuse low-grade gliomas	Pediatric-type diffuse high-grade gliomas	Circumscribed astrocytic gliomas	Glioneuronal and neuronal tumors	Ependymal tumors
Examples	Astrocytoma, IDH-mutant Oligodendroglioma, IDH-mutant and 1p/19q-codeleted GLIOBLASTOMA, IDH-wt	Diffuse astrocytoma, MYB- or MYBL1-altered Angiocentric glioma	Diffuse midline glioma, H3 K27-altered Diffuse hemispheric glioma, H3 G34-mutant	Pilocytic astrocytoma Chordoid glioma Astroblastoma, MN1-altered	Ganglioglioma Gangliocytoma Central neurocytoma	Subependymoma Spinal ependymoma, MYCN-amplified Supratentorial ependymoma, ZFTA or YAP1 fusion-positive

WHO: World Health Organization. IDH-mutant: isocitrate dehydrogenase-mutant. IDH-wt: isocitrate dehydrogenase-wild type. MN1: Meningioma 1. ZFTA: Zinc Finger Translocation Associated. YAP1: Yes-Associated Protein 1.

## Data Availability

The datasets used and/or analyzed during the current study are available from the corresponding author upon reasonable request.

## References

[1] Khabibov M., Garifullin A., Boumber Y., Khaddour K., Fernandez M., Khamitov F., Khalikova L., Kuznetsova N., Kit O., Kharin L. (2022). Signaling pathways and therapeutic approaches in glioblastoma multiforme (Review). Int. J. Oncol..

[2] Baro V., Cerretti G., Todoverto M., Della Puppa A., Chioffi F., Volpin F., Causin F., Busato F., Fiduccia P., Landi A. (2022). Newly Diagnosed Multifocal GBM: A Monocentric Experience and Literature Review. Curr. Oncol..

[3] Tan A.C., Ashley D.M., López G.Y., Malinzak M., Friedman H.S., Khasraw M. (2020). Management of glioblastoma: State of the art and future directions. CA Cancer J. Clin..

[4] Tykocki T., Eltayeb M. (2018). Ten year survival in glioblastoma. A systematic review. J. Clin. Neurosci..

[5] Pradhan P., Dey B., Srinivas B.H., Jacob S.E., Rathakrishnan R.K.V. (2018). Clinico-Histomorphological and Immunohistochemical Profile of Anaplastic Pleomorphic Xanthoastrocytoma: Report of Five Cases and Review of Literature. Int. J. Hematol. Oncol. Stem Cell Res..

[6] Grochans S., Cybulska A.M., Simińska D., Korbecki J., Kojder K., Chlubek D., Baranowska-Bosiacka I. (2022). Epidemiology of Glioblastoma Multiforme–Literature Review. Cancers.

[7] Tamimi A.F., Juweid M., De Vleeschouwer S. (2017). Epidemiology and Outcome of Glioblastoma. Glioblastoma [Internet].

[8] Pombo Antunes A.R., Scheyltjens I., Duerinck J., Neyns B., Movahedi K., Van Ginderachter J.A. (2020). Understanding the glio-blastoma immune microenvironment as basis for the development of new immunotherapeutic strategies. Elife.

[9] Brighi N., Lamberti G., Andrini E., Mosconi C., Manuzzi L., Donati G., Lisotti A., Campana D. (2023). Prospective Evaluation of MGMT-Promoter Methylation Status and Correlations with Outcomes to Temozolomide-Based Chemotherapy in Well-Differentiated Neuroendocrine Tumors. Curr. Oncol..

[10] Mir T., Pond G., Greenspoon J.N. (2021). Outcomes in Elderly Patients with Glioblastoma Multiforme Treated with Short-Course Radiation Alone Compared to Short-Course Radiation and Concurrent and Adjuvant Temozolomide Based on Performance Status and Extent of Resection. Curr. Oncol..

[11] Louis D.N., Perry A., Wesseling P., Brat D.J., Cree I.A., Figarella-Branger D., Hawkins C., Ng H.K., Pfister S.M., Reifen-berger G. (2021). The 2021 WHO Classification of Tumors of the Central Nervous System: A summary. Neuro Oncol..

[12] Hiddinga B.I., Pauwels P., Janssens A., van Meerbeeck J.P. (2017). O6-Methylguanine-DNA methyltransferase (MGMT): A druga-ble target in lung cancer?. Lung Cancer.

[13] Gozdz A. (2023). Proteasome Inhibitors against Glioblastoma—Overview of Molecular Mechanisms of Cytotoxicity, Progress in Clinical Trials, and Perspective for Use in Personalized Medicine. Curr. Oncol..

[14] Jia J.L., Alshamsan B., Ng T.L. (2023). Temozolomide Chronotherapy in Glioma: A Systematic Review. Curr. Oncol..

[15] Wei W., Chen X., Ma X., Wang D., Guo Z. (2015). The efficacy and safety of various dose-dense regimens of temozolomide for recurrent high-grade glioma: A systematic review with meta-analysis. J. Neurooncol..

[16] Raverot G., Burman P., McCormack A., Heaney A., Petersenn S., Popovic V., Trouillas J., Dekkers O.M., European Society of Endocrinology (2018). European Society of Endocrinology Clinical Practice Guidelines for the management of aggressive pituitary tumours and carcinomas. Eur. J. Endocrinol..

[17] Bengtsson D., Schrøder H.D., Andersen M., Maiter D., Berinder K., Feldt Rasmussen U., Rasmussen Å.K., Johannsson G., Hoybye C., van der Lely A.J. (2015). Long-term outcome and MGMT as a predictive marker in 24 patients with atypical pi-tuitary adenomas and pituitary carcinomas given treatment with temozolomide. J. Clin. Endocrinol. Metab..

[18] Losa M., Bogazzi F., Cannavo S., Ceccato F., Curtò L., De Marinis L., Iacovazzo D., Lombardi G., Mantovani G., Mazza E. (2016). Temozolomide therapy in patients with aggressive pituitary adenomas or carcinomas. J. Neurooncol..

[19] Campderá M., Palacios N., Aller J., Magallón R., Martín P., Saucedo G., Lilienfeld H., Estrada J. (2016). Temozolomide for ag-gressive ACTH pituitary tumors: Failure of a second course of treatment. Pituitary.

[20] Jazmati D., Hero B., Thole-Kliesch T.M., Merta J., Deubzer H.E., Bäumer C., Heinzelmann F., Schleithoff S.S., Koerber F., Eggert A. (2022). Efficacy and Feasibility of Proton Beam Therapy in Relapsed High-Risk Neuroblastoma-Experiences from the Prospective KiProReg Registry. Curr. Oncol..

[21] Lee S.Y. (2016). Temozolomide resistance in glioblastoma multiforme. Genes Dis..

[22] Singh N., Miner A., Hennis L., Mittal S. (2021). Mechanisms of temozolomide resistance in glioblastoma—A comprehensive review. Cancer Drug Resist..

[23] Banelli B., Carra E., Barbieri F., Würth R., Parodi F., Pattarozzi A., Carosio R., Forlani A., Allemanni G., Marubbi D. (2015). The histone demethylase KDM5A is a key factor for the resistance to temozolomide in glioblastoma. Cell Cycle.

[24] Kim G.W., Lee D.H., Yeon S.K., Jeon Y.H., Yoo J., Lee S.W., Kwon S.H. (2019). Temozolomide-resistant Glioblastoma Depends on HDAC6 Activity Through Regulation of DNA Mismatch Repair. Anticancer. Res..

[25] Wang Z., Hu P., Tang F., Lian H., Chen X., Zhang Y., He X., Liu W., Xie C. (2016). HDAC6 promotes cell proliferation and confers resistance to temozolomide in glioblastoma. Cancer Lett..

[26] Tiek D.M., Rone J.D., Graham G.T., Pannkuk E.L., Haddad B.R., Riggins R.B. (2018). Alterations in Cell Motility, Proliferation, and Metabolism in Novel Models of Acquired Temozolomide Resistant Glioblastoma. Sci. Rep..

[27] Jiapaer S., Furuta T., Tanaka S., Kitabayashi T., Nakada M. (2018). Potential Strategies Overcoming the Temozolomide Resistance for Glioblastoma. Neurol. Med. Chir..

[28] Stupp R., Mason W.P., van den Bent M.J., Weller M., Fisher B., Taphoorn M.J., Belanger K., Brandes A.A., Marosi C., Bogdahn U. (2005). Radiotherapy plus concomitant and adjuvant temozolomide for glioblastoma. N. Engl. J. Med..

[29] Weller M., Tabatabai G., Kästner B., Felsberg J., Steinbach J.P., Wick A., Schnell O., Hau P., Herrlinger U., Sabel M.C. (2015). MGMT Promoter Methylation Is a Strong Prognostic Biomarker for Benefit from Dose-Intensified Temozolomide Rechallenge in Progressive Glioblastoma: The DIRECTOR Trial. Clin. Cancer Res..

[30] Xiao Z.Z., Wang Z.F., Lan T., Huang W.H., Zhao Y.H., Ma C., Li Z.Q. (2020). Carmustine as a Supplementary Therapeutic Option for Glioblastoma: A Systematic Review and Meta-Analysis. Front Neurol..

[31] van den Bent M.J., Baumert B., Erridge S.C., Vogelbaum M.A., Nowak A.K., Sanson M., Brandes A.A., Clement P.M., Baurain J.F., Mason W.P. (2017). Interim results from the CATNON trial (EORTC study 26053-22054) of treatment with concurrent and adjuvant temozolomide for 1p/19q non-co-deleted anaplastic glioma: A phase 3, randomised, open-label intergroup study. Lancet.

[32] Tesileanu C.M.S., Sanson M., Wick W., Brandes A.A., Clement P.M., Erridge S.C., Vogelbaum M.A., Nowak A.K., Baurain J.F., Mason W.P. (2022). Temozolomide and Radiotherapy versus Radiotherapy Alone in Patients with Glioblastoma, IDH-wildtype: Post Hoc Analysis of the EORTC Randomized Phase III CATNON Trial. Clin. Cancer Res..

[33] Joo J.D., Kim H., Kim Y.H., Han J.H., Kim C.Y. (2015). Validation of the Effectiveness and Safety of Temozolomide during and after Radiotherapy for Newly Diagnosed Glioblastomas: 10-year Experience of a Single Institution. J. Korean Med. Sci..

[34] Wang Y., Feng Y. (2020). The efficacy and safety of radiotherapy with adjuvant temozolomide for glioblastoma: A meta-analysis of randomized controlled studies. Clin. Neurol. Neurosurg..

[35] Perry J.R., Laperriere N., O’Callaghan C.J., Brandes A.A., Menten J., Phillips C., Fay M., Nishikawa R., Cairncross J.G., Roa W. (2017). Short-Course Radiation plus Temozolomide in Elderly Patients with Glioblastoma. N. Engl. J. Med..

[36] Arvold N.D., Cefalu M., Wang Y., Zigler C., Schrag D., Dominici F. (2017). Comparative effectiveness of radiotherapy with vs. without temozolomide in older patients with glioblastoma. J. Neurooncol..

[37] Xu W., Li T., Gao L., Zheng J., Shao A., Zhang J. (2017). Efficacy and safety of long-term therapy for high-grade glioma with temozolomide: A meta-analysis. Oncotarget.

[38] Alimohammadi E., Bagheri S.R., Taheri S., Dayani M., Abdi A. (2020). The impact of extended adjuvant temozolomide in newly diagnosed glioblastoma multiforme: A meta-analysis and systematic review. Oncol. Rev..

[39] Balana C., Vaz M.A., Manuel Sepúlveda J., Mesia C., Del Barco S., Pineda E., Muñoz-Langa J., Estival A., de Las Peñas R., Fuster J. (2020). A phase II randomized, multicenter, open-label trial of continuing adjuvant temozolomide beyond 6 cycles in patients with glioblastoma (GEINO 14-01). Neuro Oncol..

[40] Gupta T., Talukdar R., Kannan S., Dasgupta A., Chatterjee A., Patil V. (2022). Efficacy and safety of extended adjuvant temozolomide compared to standard adjuvant temozolomide in glioblastoma: Updated systematic review and meta-analysis. Neurooncol. Pract..

